# Supplementary material for: Mechanisms governing the pioneering and redistribution capabilities of the non-classical pioneer PU.1
Source: Nat Commun. 2020 Jan 21;11:402. doi: 10.1038/s41467-019-13960-2 (PMC6972792; doi:10.1038/s41467-019-13960-2)
Supplement: Supplementary file 7 — Source data [file 41467_2019_13960_MOESM7_ESM.zip › Source_Data/Figure6/Figure6B_MotifScanOutput/ETS1_PU.1/homerResults/motif24.similar.html]

motif24

## Information for motif24

A
G
T
C
A
C
G
T
A
C
G
T
C
G
T
A
A
C
G
T
A
G
T
C
C
G
A
T
G
A
T
C
  
Reverse Opposite:  

C
T
A
G
C
G
T
A
A
C
T
G
C
G
T
A
A
C
G
T
C
G
T
A
C
G
T
A
T
C
A
G
  

|  |  |
| --- | --- |
| p-value: | 1e-99 |
| log p-value: | -2.294e+02 |
| Information Content per bp: | 1.812 |
| Number of Target Sequences with motif | 2715.0 |
| Percentage of Target Sequences with motif | 17.15% |
| Number of Background Sequences with motif | 3266.7 |
| Percentage of Background Sequences with motif | 10.19% |
| Average Position of motif in Targets | 99.0 +/- 55.4bp |
| Average Position of motif in Background | 100.6 +/- 64.4bp |
| Strand Bias (log2 ratio + to - strand density) | 0.1 |
| Multiplicity (# of sites on avg that occur together) | 1.13 |
| Motif File: | file (matrix) reverse opposite |

### Similar de novo motifs found

|  |  |  |  |  |  |  |  |
| --- | --- | --- | --- | --- | --- | --- | --- |
| Rank | Match Score | Redundant Motif | P-value | log P-value | % of Targets | % of Background | Motif file |
| 1 | 0.981 | A G T C A C G T A C G T C G T A A C G T A G T C C G A T | 1e-99 | -228.232605 | 17.20% | 10.24% | motif file (matrix) |
| 2 | 0.956 | A G T C A C G T C A G T C T G A A C G T A G T C C G A T T A C G G A T C | 1e-80 | -184.631478 | 9.44% | 4.81% | motif file (matrix) |
| 3 | 0.837 | A G T C A T C G G T A C A T C G T C G A T C A G C G T A G C A T T C G A C T G A | 1e-26 | -60.606501 | 4.04% | 2.26% | motif file (matrix) |
